# The effect of employment stress on employment anxiety among physical education students: the mediating role of social support and psychological resilience

**DOI:** 10.3389/fpsyg.2025.1533218

**Published:** 2025-09-04

**Authors:** Jie Wang, Ruohan Zhang

**Affiliations:** ^1^School of Physical Education, Qiqihar University, Qiqihar, China; ^2^Exercise and Sports Science Programme, School of Health Sciences, Universiti Sains Malaysia, Kubang Kerian, Kelantan, Malaysia

**Keywords:** physical education students, employment stress, social support, psychological resilience, employment anxiety

## Abstract

**Objective:**

This study aims to investigate the relationship between employment stress and employment anxiety in physical education students, especially the mediating role of social support and psychological resilience.

**Methods:**

Group psychological measurements were administered to 532 students using the Employment Stress Scale for College Students, Social Support Rating Scale (SSRS), Brief Resilience Scale (BRS), and the Questionnaire for Suppression of Career Anxiety in College Graduates, in which the data were analyzed using SPSS 26.0 and the Bootstrap method to analyze the data, test and analyze the effects.

**Results:**

Employment stress was positively related to employment anxiety, and employment stress can significantly be used to predict employment anxiety in physical education students. In addition, employment stress can play a negatively predicted role in social support and psychological resilience, in turn, both factors can also have a negative prediction role on employment anxiety at the same time. Social support and psychological resilience were identified as significant mediators between employment stress and employment anxiety with three mediating pathways: employment stress, social support and employment anxiety (Path 1), with an indirect effect of 0.122 and Bootstrap 95% confidence intervals excluding 0 (LLCI = 0.099, ULCI = 0.144), accounted for 15.20% of the total effect. Employment stress, psychological resilience, and employment anxiety (Path 2), with an indirect effect of 0.064 and Bootstrap 95% confidence intervals excluding 0 (LLCI = 0.016, ULCI = 0.115), accounting for 8.00% of the total effect. Employment Stress, Social Support, psychological resilience, employment anxiety (Path 3), with an indirect effect of 0.006 and Bootstrap 95% confidence intervals excluding 0 (LLCI = 0.002, ULCI = 0.011), accounts for 0.70% of the total effect.

**Conclusion:**

Employment stress has positively predictive effects on employment anxiety in physical education students and has a ripple effect on anxiety symptoms in physical education students through the mediating role of social support and psychological resilience. The research results show that providing social support and increasing psychological resilience can effectively improve the individual’s coping level, alleviate the impact of employment stress, and thus reduce employment anxiety, which will be beneficial to the physical and mental health development of physical education students. At the same time, it provides a theoretical basis for graduate employment guidance and employment psychology education, and better helps physical education students make career choices.

## 1 Introduction

Higher education’s employment is linked to both students’ personal futures and the growth and stability of society as a whole, serving as a link between their acquisition of professional skills and their entry into the workforce. One type of negative emotional state that arises in work-related circumstances is job anxiety. Currently, the phenomenon that college students have employment anxious is gradually spreading to younger students. Many college students are distressed by this occurrence, which has been worsen by the dire work situation. According to studies, graduates will find it more difficult to make professional selections if they have a higher level of employment anxiety, which is detrimental to their chances of finding work (Li, 2019). Some experts regard “employment anxiety” as a unique social psychological state, also known as “career anxiety.” Specifically, it includes: feeling that employment is a potential threat, having strong concerns about employment or its consequences, and long-term employment anxiety can cause some adverse physical and mental reactions, including negative emotions such as tension, fear, and physiological and behavioral reactions such as increased heart rate, chest tightness, and lack of concentration, which have a great impact on study, work and life (Wu, 2024). According to Mao and Chen (2024), this study also assists students in preventing and controlling their anxiety related to their jobs, developing a realistic professional mindset, maintaining their physical and mental well-being, and improving their personal quality of life.

Huang and Yan, 2009 defines employment stress as the psychological strain brought on by the interplay between personal cognitive assessment and objective environmental stimuli in the workplace. College students are under a lot of stress to find work because of the decrease in employment prospects and internal rivalry for talent following the epidemic (Peng et al., 2024). Approximately one-third of college students believe theyselves are under extreme employment stress, and nearly half of them experience heightened employment anxiety (Fang and Liu, 2022). Under the current socioeconomic context, the number of college graduates is rising annually, making it impossible for established jobs to meet the demands of recent graduates. This is the primary reason why sports majors confront high employment stress. They have comparatively high course learning difficulties and limited career opportunities due to the discipline attributes; therefore, their employment anxiety level deserves greater attention (Yutong and Jun, 2024).

When someone confronts hardships or emergencies, social support refers to the material and spiritual assistance and support that others offer (Pattison, 1977). One significant area for assisting students’ career development seems to be comprehending and investigating their job-finding issues (Bozgeyikli et al., 2024). Social support is a frequent protective element for mental health, and it can be given by people in good and healthy social connections. It can successfully lessen the symptoms of anxiety and sadness in individuals as well as the effects of outside stress and subjectively unfavorable emotions. In the process of employment, social support can not only help people deal with the immediate effects of stressful life events, but also encourage them to change their mindset, adopt more constructive coping strategies, and slow down the impact’s duration (Hatimah et al., 2023). Research indicates that positive social support is the external reason of employment anxiety, and an individual’s self-perception is the internal cause. Beginning with these two elements can assist people in developing a positive work attitude, lessening the stress brought on by the work environment, and approaching work with composure (Ma, 2018).

According to Cao et al. (2018), psychological resilience is a long-term adaptive skill or psychological capital that empowers people to be resilient rather than just be applied to extremely particular, one-time actions. In addition to mitigating the negative effects of unfavorable circumstances, psychological resilience exemplifies the psychological trait of fortifying oneself against hardship (Masten, 2001; Fletcher and Sarkar, 2013). In his research, Robledo discovered that even though students had positive experiences working, their mental health was somewhat harmed. This further emphasizes the significance of psychological resilience in the study of employment mental health (Robledo-Martín et al., 2023). People will activate their body’s defense mechanisms to deal with stress related to their jobs in order to keep their equilibrium. The person’s equilibrium will not be affected by modest pressure. A person will experience unpleasant emotions like worry and depression when the pressure is too great since the initial equilibrium is upset. The person can then attain a greater degree of psychological resilience and enhance their future capacity to manage stress as they reintegrate resources (Gerber et al., 2018). Because psychological resilience and job stress are closely related, improving psychological resilience may be a crucial tactic to reduce job stress (Cooper et al., 2021). Furthermore, external social support is crucial in reducing work-related stress, in addition to an individual’s internal psychological resilience (Jin and Zhang, 2021).

Specifically, this study explores how employment stress regulates employment anxiety through the mediating role of social support and psychological resilience. Based on the research results, this study will make relevant suggestions to improve the employment psychological literacy of physical education students, encourage students to pay more attention to employment issues in their thoughts and actions, and promote personal career growth.

## 2 Literature review and research hypotheses

### 2.1 The relationship between employment stress and employment anxiety

The ecological systems theory, studies pertaining to job stress and anxiety developed by U. Bronfenbrenner, which highlights how human development is nested inside a series of interdependent environmental systems. Anxiety over uncertain job prospects and a sense of helplessness over future uncertainty are the primary causes of employment anxiety among Chinese college students nowadays (Liu and Shao, 2021). Among these, the primary features of employment anxiety are the dramatic increase in unemployment and the ensuing decline in job chances (Blustein and Guarino, 2020). Many people’s careers have been severely damaged by this (Buhr and Dugas, 2002). According to research, full-time education graduates think that their work is a good indicator of their success (Carleton et al., 2007; Kim et al., 2022). But given the current situation, it is hard for young people to obtain employment, which causes unemployment rates to rise as soon as students graduate (Ji et al., 2016). The cause system of college students’ employment anxiety is made up of the macro-social work environment, the meso-social public opinion environment, the family of origin, and the micro-self situation. One of them that influences the development of workplace anxiety is employment stress, which is a person’s cognitive evaluation of the external environment’s employment stressor. In conclusion, college students’ anxiety levels increase with the amount of psychological pressure they face when making a professional decision (Turnipseed, 1992). The following hypothesis is proposed: H1—Employment anxiety is positively correlated with employment stress.

### 2.2 The relationship between employment stress, social support and employment anxiety

The link between two variables is currently the main focus of the literature that is currently available. It has been demonstrated that there is a negative correlation between college students’ social support and employment stress. Employment stress decreases when people’s sense of social support increases (Viswesvaran et al., 1999). According to research on the relationship between social support and employment anxiety, social support has a negative correlation with both the overall score of employment anxiety and each of its dimensions; in other words, the more social support an individual has, the lower their degree of employment anxiety will be (LaRocco et al., 1980). Additionally, Ashleigh J. Hillier discovered a substantial negative correlation between occupational anxiety and the degree of social support (Hillier et al., 2007). According to studies on social support and employment anxiety, college students who have a strong network of social support are also better able to handle stressful work situations and take more initiative to resolve issues like conflict, anxiety, or unfavorable feelings at work (Marcelissen et al., 1988). Thus, these findings demonstrate that social support can effectively assist students in adjusting to life after graduation and better coping with the demands of the workplace, as well as having a positive mitigating influence on job anxiety (Beehr and McGrath, 1992). In summary, it can be speculated that students with higher levels of social support are less likely to have employment anxiety. This may be because the higher the individual’s level of social support, the more proactively they can respond to different changes in society, enhance their confidence in controlling unknown factors in life, alleviate the negative emotions brought about by employment stress, and increase their confidence in finding an ideal job, thereby reducing employment anxiety. The following hypothesis is proposed: H2—Social support might act as a mediator between employment stress and employment anxiety.

### 2.3 The relationship between employment stress, psychological resilience and employment anxiety

According to previous research, those who are extremely resilient individuals think that challenges and issues are transient and can be resolved in specific situations. They may adjust more easily and employ more clever problem-solving techniques when confronted with stressful events or a changing external environment, which leads them to engage more actively in society (Garmezy, 1991; Walsh, 2003; Vella and Pai, 2019). It is challenging for people with low resilience to react flexibly and adaptively to a changing environment. They struggle to heal after trauma and are illiterate and unorganized under stress. However, people with low resilience frequently expose themselves to anxiety in settings that are unpredictable and ambiguous (Guastello, 2024). One of the main elements that controls or mediates the relationship between stress and mental health is psychological resilience, a positive psychological trait that can assist people in lessening or fending off the neg effects of stress (Harvey and Delfabbro, 2004; Sun and Yang, 2022). According to certain research, psychological resilience has a considerable negative correlation with employment anxiety and can anticipate it. Psychological resilience and employment stress are substantially correlated (Wang et al., 2017). Several survey research have demonstrated a strong inverse relationship between psychological resilience and employment anxiety, meaning that the more resilient a person is, the less symptoms of employment anxiety they will experience (Shin et al., 2019). Thus, these findings suggest that there is a strong inverse relationship between the psychological resilience of physical education students and their anxiety about their jobs; in other words, a high degree of psychological resilience can successfully lessen the stress and anxiety that physical education students face in the workplace (Xia, 2013). The following hypothesis is proposed: H3—The relationship between employment stress and employment anxiety may be mediated by psychological resilience.

### 2.4 The relationship between employment stress, social support, psychological resilience and employment anxiety

According to the investigation, social support and psychological resilience had their own mediating effects on employment stress and employment anxiety, respectively. Although no research has yet examined this particular relationship, prior studies have demonstrated a strong correlation between social support and psychological resilience (Friborg et al., 2003). This suggests that there may be a chain mediation effect in the relationship between employment stress and employment anxiety among physical education students, which has not been discussed yet. Many scholars have not explored the direction of career development from the perspective of sports majors. This article has enriched the research results of predecessors to a certain extent. One external protective component of psychological resilience is social support. It is more favorable for college students to develop psychological resilience at higher levels (Feng et al., 2016). Social support is a protective factor that has a regulatory effect on mental health and can effectively stimulate an individual’s ability to cope with various events (Shang and Yang, 2021). Psychological resilience can help people in stressful situations adopt more positive coping strategies, which can reduce perceived stress and lessen the impact of negative emotions (Nicholls et al., 2015). People may experience unfavorable emotional reactions as a result of stressful life experiences. Physical education majors’ negative emotional states and job pressure are strongly correlated; that is, the more pressure they experience, the more intense their negative emotions, including anxiety, are Yutong and Jun (2024). The subject attributes of sports majors determine the need for highly complex talents and the narrow employment direction. Physical education students not only need to combine a deep theoretical foundation with superb practical skills, but also need to stand out from the limited positions. This contradiction undoubtedly exacerbates the negative psychological impact of employment stress on individual employment anxiety of physical education students under the background of severe employment situation. Social support and psychological resilience are one of the main entry points for the development of employment guidance and psychological work in colleges and universities. Whether these two factors can play a better role in alleviating the poor psychological state of employment of physical education students needs to be explored. Therefore, this study introduces social support and psychological resilience into the psychological mechanism of employment stress and employment anxiety of physical education students, and deeply explores the internal relationship between the four variables. The following hypothesis is proposed: H4—Social support and psychological resilience may act as a chain mediation factor between employment stress and employment anxiety.

**Figure 1:**
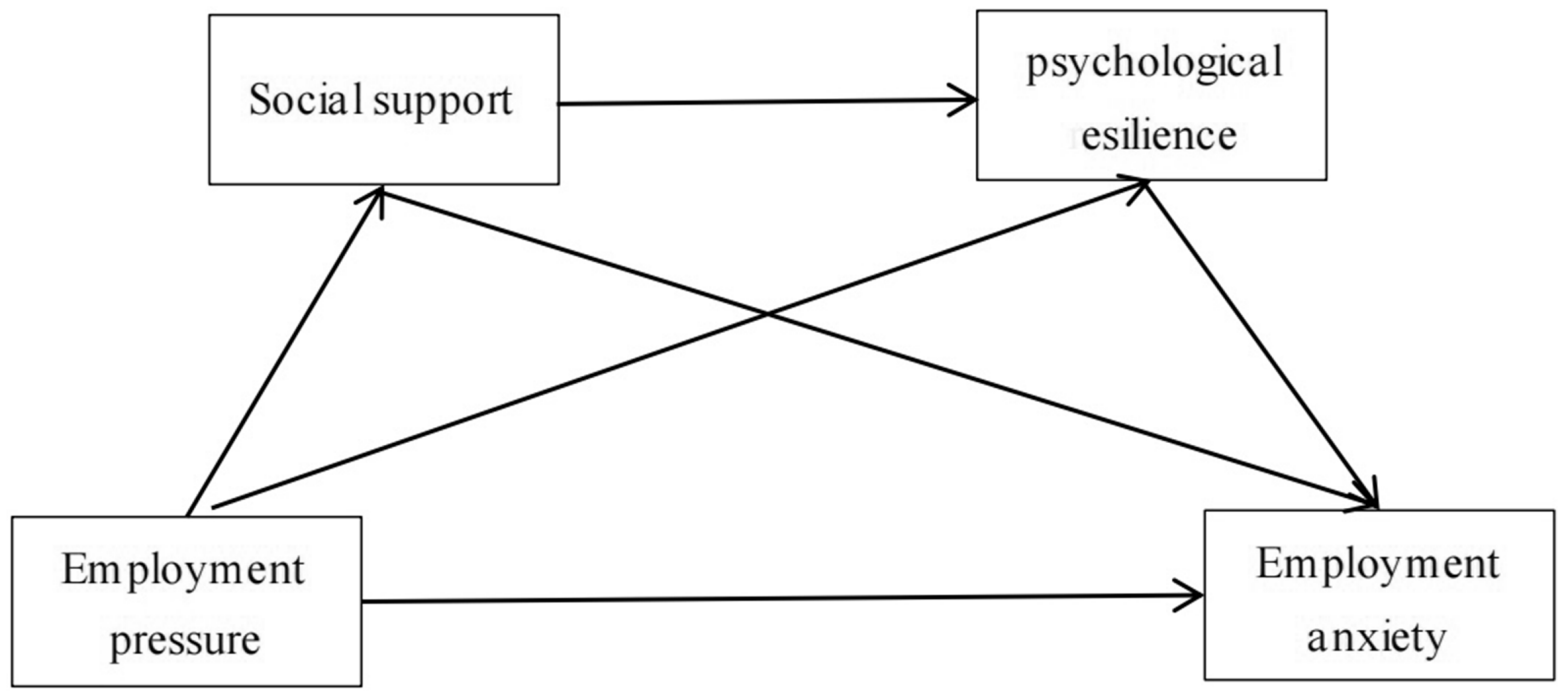
Hypothetical model of employment stress, social support, psychological resilience, and employment anxiety.

As seen in Figure 1, we developed a chain mediation model based on study hypotheses H1–H4 to investigate how employment stress affects physical education students employment anxiety by utilizing social support and psychological resilience as mediating factors.

## 3 Materials and methods

### 3.1 Participants

Five hundred and fifty physical education majors from five undergraduate colleges in Heilongjiang Province were chosen by random sampling, and questionnaires were sent out between March and May 2024. Five hundred and thirty-two valid surveys in all, with an effective rate of 96.7%, were gathered. They included 222 males (41.7%) and 310 females (58.2%); 31 freshmen (5.83%), 146 sophomores (27.44%), 180 juniors (33.84%), and 175 seniors (32.89%); 248 students from urban areas (46.62%) and 284 from rural areas (53.38%); 236 only child status (44.36%) and 296 Not an only child (55.64%). Every participant had prior Internet usage experience. Before the survey started, each participant was told of the details of the test, and their agreement was acquired.

### 3.2 Measures

#### 3.2.1 Employment stress scale for college students

Chen Yuhong updated the scale based on the 2009 employment stress scale for college students. Among its 30 components are five dimensions: professional needs, career orientation, job search assistance, employment expectations, and application quality. A five-level scoring system is used. The pressure to find work increases with a higher score (Chen, 2019). Good internal consistency was shown by the scale’s Cronbach’s alpha coefficient of 0.950 in this investigation.

#### 3.2.2 Social support rating scale (SSRS)

The scale was compiled by Xiao Shuiyuan and consists of 10 items, including 3 dimensions: objective support, subjective support and utilization of support. The scale adopts an item scoring method and uses the total score to reflect the individual’s level of social support. The higher the score, the more social support is obtained (Xiao and Yang, 1987). Since its introduction, this scale has been widely used in China and has good reliability and validity. This study calculated the Cronbach’s *α* coefficient of the social support rating scale to be 0.790, indicating that the scale has a high reliability.

#### 3.2.3 Brief resilience scale (BRS)

The Chinese version of the short-form resilience scale revised by Chen Wei et al. was used. The scale consists of 6 items and is scored on a Likert-5 point scale, with 1 representing completely disagree, 2 representing disagree, 3 representing uncertain, 4 representing agree, and 5 representing completely agree. There are 3 positive and 3 negative scoring items, and the average score reflects the individual’s psychological resilience (Chen et al., 2020). The Cronbach’s α coefficient of the Brief resilience scale in this study was 0.791, and its reliability was good.

#### 3.2.4 Career anxiety questionnaire for college graduates

The questionnaire was compiled by Zhang Yuzhu and contains 26 items covering four dimensions: employment competition pressure, lack of employment support, lack of self-confidence and concerns about employment prospects. It adopts a 5-point scoring system, where the higher the score, the more anxious the individual is (Zhang et al., 2011). The Cronbach’s α coefficient of the career anxiety questionnaire for college graduates in this study was 0.939, which has a high reliability.

#### 3.2.5 Data analyses

Excel 2019 was utilized for data entry and reverse scoring, followed by SPSS 26.0 for descriptive analysis, correlation analysis, regression analysis, and chain mediation analysis. The mediation effect analysis was carried out using the bootstrap method of the SPSS macro program Process (version 4.1) plug-in, with a specific indirect effect test model, No. 6, set as X = employment stress, M1 = social support, M2 = psychological resilience, and Y = employment anxiety; bootstrap samples were set to 5,000 times. Demographic variables of the subject group were controlled for in the stepwise regression analysis to maintain study fairness, ensuring that they were not the main focus of discussion in this article.

## 4 Results

### 4.1 Common method deviation test

Given that this study utilized self-reported questionnaires to assess the variables, there was a potential risk of common method bias. To address this, Harman’s single-factor test was employed, following the approach suggested by Zhou and Long (2004). The results show that there are a total of 13 factors with extracted eigenvalues greater than 1, and the maximum factor variance explanation rate is 38.945% (<40%). Therefore, this study does not have significant common method deviation and meets the statistical requirements.

### 4.2 Tests of difference between variables on demographic variables

The results of the difference tests are presented in Table 1. In terms of employment stress, the mean value of male is higher than that of female, and the difference is significant; the mean value of mothers’ education level is college or above, which is significantly higher than that of high school or technical secondary school and technical secondary school and below, and the difference is significant; family location, major, whether they are an only child, grade, there is no significant difference in father’s education level and economic level. In terms of social support, there were no significant differences in gender, family location, major, only child status, grade, father’s education level, mother’s education level and economic level. In terms of psychological resilience, there are no significant differences in gender, family location, major, whether an only child, grade, father’s education level, mother’s education level and economic level. In terms of employment anxiety, the mean value of senior students is higher than that of freshmen, sophomores and juniors, and the difference is significant; the mean value of mothers’ education level is college and above, which is significantly higher than high school or technical secondary school and technical secondary school and below, and the difference is significant; gender, family There were no significant differences in location, major, whether they were an only child, father’s education level and economic level.

**Table 1:** Results of the test of variability of the variables on demographic variables (*N* = 532).

Variables	Categories	Employment stress	Social support	Psychological resilience	Employment anxiety
Gender	Male	94.55 ± 21.34	32.13 ± 7.78	3.14 ± 0.72	74.58 ± 18.71
	Female	88.51 ± 28.29	31.27 ± 7.71	3.23 ± 0.95	72.10 ± 25.09
	*t*	2.804	1.252	−2.172	1.302
	*P*	0.000**	0.211	0.000**	0.000**
Family location	Urban area	91.00 ± 26.29	31.85 ± 7.83	3.25 ± 0.87	72.94 ± 22.96
	Rural area	91.05 ± 25.35	31.44 ± 7.68	3.21 ± 0.86	73.31 ± 22.44
	*t*	−0.02	0.615	0.519	−0.19
	*P*	0.984	0.539	0.604	0.849
Major	Discipline	90.70 ± 27.44	30.92 ± 7.69	3.21 ± 0.94	73.86 ± 24.61
	Technical	91.55 ± 22.92	32.76 ± 7.71	3.27 ± 0.72	71.98 ± 19.15
	*t*	−0.382	−2.683	−0.758	0.985
	*P*	0.000**	0.008	0.000**	0.000**
The only child in the family	Yes	92.42 ± 23.85	32.11 ± 7.84	3.22 ± 0.84	73.85 ± 20.86
	No	89.92 ± 27.19	31.25 ± 7.66	3.24 ± 0.89	72.56 ± 24.02
	*t*	1.129	1.279	−0.292	0.661
	*P*	0.000**	0.202	0.770	0.509*
Grade	Freshman	91.68 ± 20.99	31.87 ± 7.60	3.06 ± 0.72	72.10 ± 17.54
	Sophomore	90.75 ± 25.36	32.07 ± 7.42	3.27 ± 0.90	72.74 ± 22.65
	Junior	86.83 ± 30.36	30.79 ± 7.74	3.32 ± 1.00	71.75 ± 26.91
	Senior	95.46 ± 20.70	32.08 ± 8.02	3.13 ± 0.68	75.07 ± 18.30
	*F*	3.453	1.062	2.173	0.798
Father’s education level	Technical secondary school and below	89.77 ± 27.82	30.95 ± 7.83	3.23 ± 0.95	73.25 ± 24.94
	High school or technical secondary school	90.65 ± 26.45	31.64 ± 7.76	3.26 ± 0.87	73.16 ± 23.06
	University and above	94.14 ± 19.63	32.93 ± 7.44	3.18 ± 0.64	72.88 ± 16.69
	*F*	1.547	2.452	0.422	0.014
Mother’s education level	Technical secondary school and below	88.05 ± 28.23	31.30 ± 7.82	3.27 ± 0.97	71.22 ± 25.34
	High school or technical secondary school	92.26 ± 24.66	31.71 ± 7.53	3.23 ± 0.82	74.43 ± 21.34
	University and above	96.11 ± 19.99	32.31 ± 8.04	3.14 ± 0.65	75.26 ± 17.24
	*F*	4.218	0.571	0.948	1.613
Economic level	General	89.31 ± 28.21	31.20 ± 7.69	3.25 ± 0.95	72.28 ± 24.85
	Medium	91.65 ± 24.80	31.48 ± 8.09	3.24 ± 0.83	74.46 ± 22.12
	Better	94.42 ± 19.99	33.11 ± 7.02	3.15 ± 0.66	72.67 ± 16.85
	*F*	1.715	2.082	0.721	0.526

*p < 0.05, **p < 0.01, ***p < 0.001.

### 4.3 Correlation between employment stress, social support, psychological resilience and employment anxiety

Pearson correlation analysis was used to analyze the correlation between the four variables of employment stress, social support, psychological resilience and employment anxiety of the physical education students participating in this study. The results found (Table 2) that the employment stress and employment anxiety of the physical education students participating in this study were significantly negatively correlated with social support and psychological resilience (*p* < 0.01). There is a significant positive correlation between employment stress and employment anxiety, social support and psychological resilience (*p* < 0.01). This provides the premise and basis for further testing the mediating role of social support and psychological resilience between employment stress and employment anxiety.

**Table 2:** Correlation analysis results of employment stress, social support, psychological resilience, and employment anxiety (*n* = 532).

Variable	M ± SD	Employment stress	Social support	Psychological resilience	Employment anxiety
Employment stress	91.03 ± 25.772	1			
Social support	31.63 ± 7.744	−0.478**	1		
Psychological resilience	3.23 ± 0.864	−0.848**	0.523**	1	
Employment anxiety	73.14 ± 22.662	0.900**	−0.655**	−0.822**	1

**p < 0.01, the correlation coefficient was significant.

### 4.4 Mediating effect analysis

Taking the employment stress of sports major students as the independent variable, employment anxiety as the dependent variable, social support and psychological resilience as the mediating variables, this study used SPSS26.0 and PROCESS4.1 plug-ins to analyze the relationship between the factors under the condition of controlling demographic variables such as gender, age, place of origin, and major category, and examined the chain mediating role of social support in the relationship between employment stress and employment anxiety. The results are shown in Table 3. The analysis includes several steps. First, a regression model is established using employment anxiety as the dependent variable and employment stress as the independent variable. The results show that employment stress has a significant positive impact on employment anxiety (*β* = 0.803, *p* < 0.001), which supports Research hypothesis 1. As can be seen from the table above, the employment stress of the sports major students participating in this study positively predicted employment anxiety (*β* = 0.803, *p* < 0.001) and negatively predicted social support (*β* = −0.154, *p* < 0.001); When employment stress and social support predicted psychological resilience at the same time, employment stress negatively predicted psychological resilience (β = −0.026, p < 0.001), and social support positively predicted psychological resilience (*β* = 0.016, *p* < 0.001); When employment stress, social support, and psychological resilience were simultaneously entered into the equation, social support negatively predicted employment anxiety (*β* = −0.788, *p* < 0.001), psychological resilience negatively predicted employment anxiety (*β* = −2.427, *p* < 0.05), and employment stress positively predicted employment anxiety (*β* = 0.611, *p* < 0.001). The regression coefficients show that social support and psychological resilience play a partial mediating role and a chain mediating role in employment stress and employment anxiety, supporting Hypothesis 2 and Hypothesis 3.

**Table 3:** Mediating effect regression model of social support and psychological resilience.

	Employment anxiety		Social support		Psychological resilience		Employment anxiety	
	*β*	*t*	*β*	*t*	*β*	*t*	*β*	*t*
Employment stress	0.803	48.213***	−0.154	−13.591***	−0.026	−29.594***	0.611	23.483***
Social support					0.016	5.430***	−0.788	−14.545***
Psychological resilience							−2.447	−3.122**
*R*	0.905		0.531		0.86		0.938	
R2	0.819		0.282		0.74		0.879	
*F*	263.118***		22.778***		147.882***		343.314***	

**p < 0.05, the regression coefficient is significant. ***p < 0.001, the regression coefficient is significant.

Through 5,000 bootstrap tests, the mediating effects and confidence intervals of social support and psychological resilience in the relationship between employment stress and employment anxiety were examined (see Table 4). The results show that the total effect of employment stress on employment anxiety is 0.803, and the 95% confidence interval of the mediating effect of social support and psychological resilience (LLCI = 0.771, ULCI = 0.836) is not 0, indicating that employment stress has a significant impact on employment anxiety. The total effect and mediating effect of social support and psychological resilience were statistically significant. The direct effect of employment stress on employment anxiety is 0.611, and the guided 95% confidence interval does not include 0 (LLCI = 0.560, ULCI = 0.662), indicating that the direct effect of employment stress on employment anxiety is significant, accounting for 76.10% of the total effect.

**Table 4:** Mediating effects of social support and psychological resilience.

		Effect	Boot SE	Boot LLCI	Boot ULCI	Ratio of indirect to total effect
Total effect		0.803	0.017	0.771	0.836	
Direct effect		0.611	0.026	0.56	0.662	0.761
	Total indirect effect	0.192	0.029	0.136	0.251	0.239
Indirect effect	Indirect effect 1 (MI-Y)	0.122	0.011	0.099	0.144	0.152
	Indirect effect 2 (M2-Y)	0.064	0.025	0.016	0.115	0.08
	Indirect effect 3 (MI-M2-Y)	0.006	0.002	0.002	0.011	0.007
	Compare 1	0.057	0.028	0.001	0.112	
	Compare 2	0.116	0.012	0.094	0.139	
	Compare 3	0.058	0.023	0.015	0.104	

**Figure 2:**
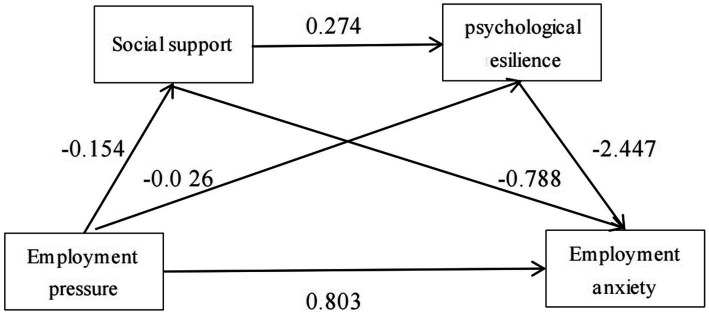
Chain mediation effect model of social support and psychological resilience. ***p < 0.001, the path coefficient was significant.

This study found that employment stress can predict the employment anxiety level of sports students, and social support and psychological resilience play an indirect mediating role through three different paths, as shown in Figure 2. The total indirect effect was calculated to be 0.192, and the Bootstrap 95% confidence interval did not include 0 (LLCI = 0.136 to ULCI = 0.251), accounting for 23.90% of the total effect. Employment stress-social support-employment anxiety (Path 1), showed an indirect effect of 0.122, and the Bootstrap 95% confidence interval did not include 0 (LLCI = 0.099, ULCI = 0.144), accounting for 15.20% of the total effect. Employment stress-psychological resilience-employment anxiety (path 2) showed an indirect effect of 0.064, and the Bootstrap 95% confidence interval did not contain 0 (LLCI = 0.016, ULCI = 0.115), accounting for 8.00% of the total effect. The chain mediation of employment stress-social support-psychological resilience-employment anxiety (path 3) has an indirect effect of 0.006, and the Bootstrap 95% confidence interval does not contain 0 (LLCI = 0.002, ULCI = 0.011), accounting for 0.70% of the total effect. These results support research hypothesis H4. Based on this, this paper proposes a chain mediation model between social support and psychological resilience in physical education students and employment stress and anxiety.

## 5 Discussion

### 5.1 The direct impact of employment stress on employment anxiety of students majoring in physical education

In line with other research, the pertinent findings of this study demonstrate that employment stress can considerably and favorably predict employment anxiety (Moxham et al., 2018). Due to the presence of stimuli, people in the dynamic process of stress evaluate them cognitively in various ways, which leads to the production of both mental and physical reactions (Sun et al., 2021). As an external stimulation, people will view their jobs as a significant aspect of their life from both their own and their immediate surroundings’ (family, school, and society) points of view. Furthermore, people’s poor perceptions of their employment position have been made worse by the highly competitive job market, which has resulted in negative emotional and physical responses (like worry). During this phase, people can successfully lessen the negative effects of job pressure on themselves if they can embrace healthy coping mechanisms. Put differently, constructive coping strategies operate as buffers against the negative effects of work-related stress on a person’s mental health. As a student majoring in physical education, they can use sports to alleviate the negative emotions of employment anxiety. On the one hand, it can help regulate bad psychological states, and on the other hand, it can also improve the technical level of athletic ability.

### 5.2 The mediating role of social support in the relationship between employment stress and employment anxiety among students majoring in physical education

Employment stress, social support, and employment anxiety are significantly correlated, according to the study’s pertinent findings. According to Hillier et al. (2011), social support decreases and employment anxiety increases with increasing job pressure. The mediation test’s findings indicate that social support somewhat mediates the relationship between job stress and job anxiety. As the current work environment has changed, employment stress has progressively grown to be a difficult and dangerous element for people, ultimately becoming a stressor. It increases the need for personal adaptation. People who are unable to cope will experience negative emotional and physical responses, which will impact their development and overall health (Milner et al., 2016). Social support theory states that a person’s ability to handle obstacles and failures will improve with their level of social support. A strong social network might be protective when it comes to managing work-related stress. It provides emotional support in order to preserve a person’s health and control their immunological or neuroendocrine systems. Additionally, it can successfully govern a person’s behavior, uphold a positive and healthy outlook on life, and improve self-control, all of which contribute to the maintenance of both physical and mental health (Sirin et al., 2013). Effective social support helps physical education students to face their career choices more calmly, which is a good emotional experience for students who have just entered the society to start their careers. Social support plays a guiding role. It can not only reduce the emotional response of employment stress to employment anxiety, but also call on more students to join the sports major, actively develop sports disciplines, and enrich the number of physical education students employed in the workplace, thereby continuously improving the national physical fitness level.

### 5.3 The mediating role of psychological resilience in the relationship between employment stress and employment anxiety among students majoring in physical education

The pertinent findings of this study demonstrate a considerable correlation between psychological resilience, employment anxiety, and employment stress. Psychological resilience decreases and employment anxiety increases with increasing employment stress. According to the findings of the mediation test, psychological resilience partially mediates the relationship between employment stress and employment anxiety (Ge et al., 2020). The impact of the epidemic has made it more difficult for students majoring in sports to obtain certification, find employment, intern, etc., even if various employment policies have shifted in favor of the sports industry in recent years. Some students now have unfavorable expectations for their careers as a result of this. They face subjective employment barriers in addition to the uncertainty of their own career planning, which has significantly impeded both the sports industry and the healthy development of individuals (Mimoun et al., 2020; Li, 2021). Better psychologically resilient people are able to handle life’s challenges and setbacks with a more upbeat outlook and more hopeful expectations about the potential consequences, which can significantly lessen the detrimental effects of pressure from the workplace on people. In a similar vein, those with high psychological resilience report less stress at work than people with low psychological resilience, and they can recover more quickly even in the same stressful situation (Steensma et al., 2007). Improving psychological toughness is the most direct and effective means for physical education students. The reason is that the learning process of sports requires practitioners to undergo long-term physical training, skill training, and experience in grades and rankings. The characteristics of the sports major strengthen the variable of psychological resilience, which undoubtedly enables physical education students to play a positive role in regulating their mental health through the “athlete spirit” when facing employment stress and employment anxiety.

### 5.4 The chain mediating effect of social support and psychological resilience on employment stress and employment anxiety among students majoring in physical education

The results of the chain mediation test confirm the chain mediation effect of psychological resilience and social support by demonstrating that the combined effect of these two factors partially explains the influence of employment stress on employment anxiety. In other words, physical education students employment stress might have a direct impact on their anxiety levels, as well as an indirect one through psychological resilience and social support. Individual social support and psychological resilience will decline as employment stress increases. According to Mohanty (2010), people who have greater psychological resilience and more social support are more likely to find meaning in their lives and be able to see the positive aspects of their current situation. This encourages them to use more constructive coping mechanisms to lessen the negative effects of work-related stress on themselves. While job resources (social support, psychological resilience) can have a positive impact by improving job engagement (the degree of integration into the job), and job demands (employment stress) will continuously consume people’s energy and cause burnout, which will eventually have a negative impact, according to the Job Demands and Resources Model (JD-R) (Demerouti et al., 2001). Job resources can operate as a buffer against high job demands; that is, they can lessen the detrimental effects that high job demands have on people. This could offer an additional perspective on how psychological resilience and social support contribute to employment stress and anxiety. The results of the chain mediation test can help provide reference for students of other majors. There are mainly two aspects. On the one hand, the narrow range of employment options for physical education students can remind and inspire students of other majors. For example: actively seek social support, pay attention to career choice trends in advance, obtain relevant qualifications, promote the development of individual multi-faceted abilities, etc., and make preparations for employment in advance when choosing limited jobs. On the other hand, physical education students have long-term professional skills learning to form positive qualities such as optimism, cheerfulness, good communication skills, and strong ability to accept setbacks, which can guide students of other majors to participate in sports, enrich sports social activities, improve stress resistance, and thus improve psychological resilience, effectively alleviating employment stress and employment anxiety.

## 6 Conclusion and proposal

### 6.1 Conclusion

Students majoring in sports had their employment anxiety, psychological resilience, social support, and employment stress examined by a questionnaire survey. The findings indicate that while current physical education students have decent levels of psychological resilience and social support, they typically experience high levels of occupational stress and anxiety. While social support and psychological resilience negatively predict employment anxiety, employment stress favorably predicts it. Social support and psychological resilience are adversely correlated with employment stress, and employment anxiety and social support and psychological resilience mediate each other in a cascade. This implies that by strengthening people’s psychological resilience and social support, we can improve their capacity to adjust to stressful work environments and lessen the detrimental effects of job anxiety.

### 6.2 Proposal

The study’s findings demonstrate that psychological resilience and strong social support might lessen the stress and anxiety associated with the workplace. Therefore, in order to help college students who are majoring in sports better transition into the workforce, the state, society, families, and educational institutions should collaborate to provide their mental health thorough attention. Parents should lower their expectations and consider their children’s employment issues in a gentle mood; education administrative departments should modify the policy on mental health education for college students in sports colleges based on the characteristics of sports students; schools should set up a comprehensive career planning mechanism; and relevant administrative departments should develop pertinent policies based on the characteristics of sports students.

## 7 Limitations of the study

Although this work has theoretical and practical implications, it still has certain limits and requires future development. Initially, this study’s survey participants are restricted to Heilongjiang Province’s sports colleges. Therefore, there are certain limitations in the selection of students in the region as participants in the research. Second, the data of this study came from subjective questionnaire tests of the subjects and lacked objective tests, which could be supplemented in the research in the future. Last but not least, this study employed a cross-sectional research design to investigate the relationship between employment stress, social support, psychological resilience, and employment anxiety; in the future, longitudinal or experimental research methods can be used to further verify the results of this study.

## Data Availability

The original contributions presented in the study are included in the article/supplementary material, further inquiries can be directed to the corresponding author/s.

## References

[ref1] Beehr T. A. McGrath J. E. (1992). Social support, occupational stress and anxiety. Anxiety Stress Coping 5, 7–19. doi: 10.1080/10615809208250484

[ref2] Blustein D. L. Guarino P. A. (2020). Work and unemployment in the time of COVID-19: the existential experience of loss and fear. J. Humanist. Psychol. 60, 702–709. doi: 10.1177/0022167820934229

[ref3] Bozgeyikli H. Görgülü Z. Boğazlıyan E. E. (2024). “Not joblessness but a job I dislike scares me”: exploring employment anxiety. Ahmet Keleşoğlu Eğitim Fakültesi Dergisi 6, 16–29. doi: 10.38151/akef.2024.127

[ref4] Buhr K. Dugas M. J. (2002). The intolerance of uncertainty scale: psychometric properties of the English version. Behav. Res. Ther. 40, 931–945. doi: 10.1016/S0005-7967(01)00092-4 12186356

[ref5] Cao N. Yan S. Zhu J. Liu Y. Liu C. Liu L. (2018). Correlations between anxiety and depression, and mental elasticity in malignant Hematopathy patients. Med One 3. doi: 10.20900/mo.20180006

[ref6] Carleton R. N. Norton M. P. J. Asmundson G. J. (2007). Fearing the unknown: a short version of the intolerance of uncertainty scale. J. Anxiety Disord. 21, 105–117. doi: 10.1016/j.janxdis.2006.03.014, PMID: 16647833

[ref7] Chen Y. (2019). Revision of the employment stress scale for college students. Educ. Modernization 6, 162–165. doi: 10.16541/j.cnki.2095-8420.2019.42.056

[ref8] Chen W. Jie L. Jie L. Guoqing L. (2020). Reliability and validity test of the short-form resilience scale among Chinese college students. Chin. J. Clin. Psych. 28, 24–28. doi: 10.16128/j.cnki.1005-3611.2020.01.006

[ref9] Cooper A. L. Brown J. A. Leslie G. D. (2021). The impact of organisational values on nurse resilience: a mixed-methods study. J. Nurs. Manag. 29, 2074–2083. doi: 10.1111/jonm.13338, PMID: 33856073

[ref10] Demerouti E. Bakker A. B. Nachreiner F. Schaufeli W. B. (2001). The job demands-resources model of burnout. J. Appl. Psychol. 86, 499–512. doi: 10.1037/0021-9010.86.3.499 11419809

[ref11] Fang X. Liu J. (2022). The current situation and influencing factors of college students’ employment anxiety during the epidemic. China Employment. 2, 46–47. doi: 10.16622/j.cnki.11-3709/d.2022.02.019

[ref12] Feng Z. Pengyu W. Qin H. Ni H. X. Mingjin X. Xinguo Y. (2016). The relationship between social support, resilience, cyber-bullying and life satisfaction among college students. Chin. J. Health Educ. 32:31. doi: 10.16168/j.cnki.issn.1002-9982.2016.01.002

[ref13] Fletcher D. Sarkar M. (2013). Psychological resilience: a review and critique of definitions, concepts, and theory. Eur. Psychol. 18, 12–23. doi: 10.1027/1016-9040/a000124

[ref14] Friborg O. Hjemdal O. Rosenvinge J. H. Martinussen M. (2003). A new rating scale for adult resilience: what are the central protective resources behind healthy adjustment? Int. J. Methods Psych. Res. 12, 65–76. doi: 10.1002/mpr.143, PMID: 12830300 PMC6878238

[ref15] Garmezy N. (1991). Resiliency and vulnerability to adverse developmental outcomes associated with poverty. Am. Behav. Sci. 34, 416–430. doi: 10.1177/0002764291034004003

[ref16] Ge J. Yang J. Song J. Jiang G. Zheng Y. (2020). Dispositional mindfulness and past-negative time perspective: the differential mediation effects of resilience and inner peace in meditators and non-meditators. PRBM 13, 397–405. doi: 10.2147/PRBM.S229705, PMID: 32440239 PMC7212969

[ref17] Gerber M. Best S. Meerstetter F. Walter M. Ludyga S. Brand S.. (2018). Effects of stress and mental toughness on burnout and depressive symptoms: a prospective study with young elite athletes. J. Sci. Med. Sport 21, 1200–1205. doi: 10.1016/j.jsams.2018.05.018, PMID: 29859672

[ref18] Guastello S. J. (2024). Elasticity, rigidity, and resilience in occupational contexts. Nonlinear Dynam. Psychol. Life Sci. 28, 389–408, PMID: 38880500

[ref19] Harvey J. Delfabbro P. (2004). Psychological resilience in disadvantaged youth: a critical overview. Aust. Psychol. 39, 3–13. doi: 10.1080/00050060410001660281

[ref20] Hatimah N. A. Dewi E. M. P. Halima A. (2023). Relationship between parents’ social support and anxiety in facing the world of work in fresh graduate. KnE Soc. Sci., 228–241. doi: 10.18502/kss.v8i19.14368

[ref21] Hillier A. Campbell H. Mastriani K. Izzo M. V. Kool-Tucker A. K. Cherry L.. (2007). Two-year evaluation of a vocational support program for adults on the autism Spectrum. Career Develop. Except. Individuals 30, 35–47. doi: 10.1177/08857288070300010501

[ref22] Hillier A. J. Fish T. Siegel J. H. Beversdorf D. Q. (2011). Social and vocational skills training reduces self-reported anxiety and depression among young adults on the autism Spectrum. J. Dev. Phys. Disabil. 23, 267–276. doi: 10.1007/s10882-011-9226-4

[ref23] Huang X. Yan W. (2009). A review of the research on the relationship between employment pressure and employment anxiety among college students. China Educ. Innov. Herald 6:8. Available at: https://kns.cnki.net/KCMS/detail/detail.aspx?dbcode=CJFN&dbname=CJFDN0911&filename=GWDT200934006 (Accessed November 5, 2024).

[ref24] Ji K.-Y. Han J.-Y. (2016). A study on the comparative study for the four-year collegiate career preparation behavior by grade level: the case of C university. J. Digital Convergence 14, 33–41.

[ref25] Jin X. Zhang L. (2021). Research on employment psychological pressure and coping ability improvement of college graduates from the perspective of psychological resilience. J. Qiannan Norm. Univ. National. 41, 86–90.

[ref26] Kim J. Oh J. Rajaguru V. (2022). Job-seeking anxiety and job preparation behavior of undergraduate students., in Healthcare, (MDPI. Available online at: https://www.mdpi.com/2227-9032/10/2/288 (Accessed October 27, 2024). 10.3390/healthcare10020288 PMC8872297 35206902

[ref27] LaRocco J. M. House J. S. French J. R. Jr. (1980). Social support, occupational stress, and health. J. Health Soc. Behav. 21, 202–218. doi: 10.2307/2136616 7410798

[ref28] Li Jiagen (2019). The relationship between career anxiety, career decision-making self-efficacy and career decision-making difficulties. Liaoning Normal University. Available online at: https://kns.cnki.net/KCMS/detail/detail.aspx?dbcode=CMFD&dbname=CMFD201902&filename=1019053167.nh (Accessed November 5, 2024)

[ref29] Li Z. (2021). A brief analysis of employment difficulties and countermeasures for college students against the background of COVID-19 pandemic., in 1st international conference on education: Current issues and digital technologies (ICECIDT 2021), (Atlantis press). Available online at: https://www.atlantis-press.com/proceedings/icecidt-21/125957224 (Accessed November 3, 2024).

[ref30] Liu S. Huang J. L. Wang M. (2014). Effectiveness of job search interventions: a meta-analytic review. Psychol. Bull. 140, 1009–1041. Available at: https://psycnet.apa.org/fulltext/2014-07946-001.html (Accessed October 27, 2024)., PMID: 24588365 10.1037/a0035923

[ref31] Liu S. Shao T. (2021). Investigation and preventive study on anxiety disorder of postgraduates under employment pressure. Psychiatr. Danub. 33, 458–459. Available at: https://hrcak.srce.hr/file/390215 (Accessed October 26, 2024).

[ref32] Ma Lifang (2018). Analysis on the structure, development characteristics and related influencing factors of employment anxiety among secondary vocational students. Liaoning Normal University. Available online at: https://kns.cnki.net/KCMS/detail/detail.aspx?dbcode=CMFD&dbname=CMFD201802&filename=1018176978.nh (Accessed November 5, 2024)

[ref33] Mao Y. Chen I.-H. (2024). The relationship between employment pressure and anxiety among art COLLEGE students at s university in HENAN Province. Available online at: https://conference.stamford.edu/wp-content/uploads/2024/09/125.pdf (Accessed October 26, 2024).

[ref34] Marcelissen F. H. Winnubst J. A. Buunk B. de Wolff C. J. (1988). Social support and occupational stress: a causal analysis. Soc. Sci. Med. 26, 365–373. doi: 10.1016/0277-9536(88)90402-9, PMID: 3347857

[ref35] Masten A. S. (2001). Ordinary magic: resilience processes in development. Am. Psychol. 56, 227–238. doi: 10.1037/0003-066X.56.3.227, PMID: 11315249

[ref36] Milner A. Krnjacki L. Butterworth P. LaMontagne A. D. (2016). The role of social support in protecting mental health when employed and unemployed: a longitudinal fixed-effects analysis using 12 annual waves of the HILDA cohort. Soc. Sci. Med. 153, 20–26. doi: 10.1016/j.socscimed.2016.01.050, PMID: 26867208

[ref37] Mimoun E. Ben Ari A. Margalit D. (2020). Psychological aspects of employment instability during the COVID-19 pandemic. Psychol. Trauma Theory Res. Pract. Policy 12, S183–S185. doi: 10.1037/tra0000769, PMID: 32538650

[ref38] Mohanty M. S. (2010). Effects of positive attitude and optimism on employment: evidence from the US data. J. Socio-Econ. 39, 258–270. doi: 10.1016/j.socec.2009.12.004

[ref39] Moxham L. J. Fernandez R. Kim B. Lapkin S. ten Ham-Baloyi W. Al Mutair A. (2018). Employment as a predictor of mental health, psychological distress, anxiety and depression in Australian pre-registration nursing students. J. Prof. Nurs. 34, 502–506. doi: 10.1016/j.profnurs.2018.03.005, PMID: 30527700

[ref40] Nicholls A. R. Perry J. L. Jones L. Sanctuary C. Carson F. Clough P. J. (2015). The mediating role of mental toughness in sport. J. Sports Med. Phys. Fitness 55, 824–834, PMID: 26360967

[ref41] Pattison E. M. (1977). A theoretical-empirical base for social system therapy. Current perspectives in cultural psychiatry. New York: Spectrum, 217–253.

[ref42] Peng Y. Lv S. B. Low S. R. Bono S. A. (2024). The impact of employment stress on college students: psychological well-being during COVID-19 pandemic in China. Curr. Psychol. 43, 18647–18658. doi: 10.1007/s12144-023-04785-w, PMID: 37359658 PMC10228455

[ref43] Robledo-Martín J. Acea-López L. Pérez-Urdiales I. Alcolea-Cosín M. T. Bellon F. Oter-Quintana C.. (2023). From students to nurses under pressure: nursing students’ entry into employment during the first COVID-19 wave. J. Clin. Nurs. 32, 7209–7226. doi: 10.1111/jocn.16800, PMID: 37335081

[ref45] Shang Y. Yang S.-Y. (2021). The effect of social support on athlete burnout in weightlifters: the mediation effect of mental toughness and sports motivation. Front. Psychol. 12:649677. doi: 10.3389/fpsyg.2021.649677, PMID: 34054652 PMC8163221

[ref46] Shin Y.-C. Kim S. M. Kim H. Min K. J. Yoo S.-K. Kim E.-J.. (2019). Resilience as a protective factor for depressive mood and anxiety among Korean employees. J. Korean Med. Sci. 34:e188. doi: 10.3346/jkms.2019.34.e188, PMID: 31293112 PMC6624414

[ref47] Sirin S. R. Gupta T. Ryce P. Katsiaficas D. Suárez-Orozco C. Rogers-Sirin L. (2013). Understanding the role of social support in trajectories of mental health symptoms for immigrant adolescents. J. Appl. Dev. Psychol. 34, 199–207. doi: 10.1016/j.appdev.2013.04.004

[ref48] Steensma H. Heijer M. D. Stallen V. (2007). Research note: effects of resilience training on the reduction of stress and depression among Dutch workers. Int. Q. Community Health Educ. 27, 145–159. doi: 10.2190/IQ.27.2.e, PMID: 18364303

[ref49] Sun W. Wang N. Shen L. (2021). The relationship between employment pressure and occupational delay of gratification among college students: positive psychological capital as a mediator. Curr. Psychol. 40, 2814–2819. doi: 10.1007/s12144-019-00209-w

[ref50] Sun X. Yang X. (2022). The structure of mental elasticity education for children in plight using deep learning. Front. Psychol. 12:766658. doi: 10.3389/fpsyg.2021.766658, PMID: 35273532 PMC8902162

[ref51] Turnipseed D. (1992). Anxiety and perceptions of the work environment. J. Soc. Behav. Pers. 7:375.

[ref52] Vella S.-L. C. Pai N. B. (2019). A theoretical review of psychological resilience: defining resilience and resilience research over the decades. Archives Med. Health Sci. 7, 233–239. doi: 10.4103/amhs.amhs_119_19

[ref53] Viswesvaran C. Sanchez J. I. Fisher J. (1999). The role of social support in the process of work stress: a meta-analysis. J. Vocat. Behav. 54, 314–334. doi: 10.1006/jvbe.1998.1661

[ref54] Walsh F. (2003). Family resilience: a framework for clinical practice. Fam. Process 42, 1–18. doi: 10.1111/j.1545-5300.2003.00001.x, PMID: 12698595

[ref55] Wang Y. W. Liu G. Z. Zhou X. T. Sheng P. J. Cui F. F. Shi T. (2017). Mediating effect of mental elasticity on occupational stress and depression in female nurses. Zhonghua lao dong wei Sheng zhi ye Bing za zhi= Zhonghua Laodong Weisheng Zhiyebing Zazhi= Chinese Journal of industrial hygiene and occupational diseases Available online at: https://europepmc.org/article/med/28780820 (Accessed October 27, 2024). 10.3760/cma.j.issn.1001-9391.2017.06.009 28780820

[ref56] Wu Zhiying (2024). The psychological mechanism of employment stress on employment anxiety and intervention of college students. Guangzhou university: The psychological mechanism of employment stress on employment anxiety and intervention of college students

[ref57] Xia Qiang (2013). Study on the survey of current situ ation of college students’ resilience and its educational countermeasures. Chengdu University of Technology. Available online at: https://kns.cnki.net/KCMS/detail/detail.aspx?dbcode=CMFD&dbname=CMFD201301&filename=1012500913.nh (Accessed November 3, 2024).

[ref58] Xiao S. Yang D. (1987). The impact of social support on physical and mental health. Chin. Ment. Health J., 183–187.

[ref59] Yutong M. Jun L. (2024). A study on the employment intentions and influencing factors of sports education major students. Advances Vocat. Technic. Educ. 6, 1–10. Available at: https://www.clausiuspress.com/assets/default/article/2024/01/19/article_1705720123.pdf (Accessed November 3, 2024).

[ref60] Zhang Y. Dewen Y. (2011). Cross-regional validation and comparison of the career anxiety questionnaire for college graduates. Psychol. Behav. Res. 9, 120–124. Available online at: https://kns.cnki.net/KCMS/detail/detail.aspx?dbcode=CJFQ&dbname=CJFD2011&filename=CLXW201102010 (Accessed November 3, 2024).

[ref61] Zhou H. Long L. (2004). Statistical remedies for common method biases. Adv. Psychol. Sci. 12:942. Available online at: https://journal.psych.ac.cn/adps/EN/Y2004/V12/I06/942

